# Detection of Multiple Innervation Zones from Multi-Channel Surface EMG Recordings with Low Signal-to-Noise Ratio Using Graph-Cut Segmentation

**DOI:** 10.1371/journal.pone.0167954

**Published:** 2016-12-15

**Authors:** Hamid Reza Marateb, Morteza Farahi, Monica Rojas, Miguel Angel Mañanas, Dario Farina

**Affiliations:** 1 The Biomedical Engineering Department, Engineering Faculty, the University of Isfahan, Isfahan, Iran; 2 Department of Automatic Control, Biomedical Engineering Research Center, Universitat Politècnica de Catalunya. BarcelonaTech (UPC), Barcelona, Spain; 3 Department of Bioengineering, Universidad El Bosque, Bogotá, Colombia; 4 Department of NeuroRehabilitation Engineering, Bernstein Center for Computational Neuroscience, University Medical Center Göttingen, Georg-August University, Göttingen, Germany; University of Sydney, AUSTRALIA

## Abstract

Knowledge of the location of muscle Innervation Zones (IZs) is important in many applications, e.g. for minimizing the quantity of injected botulinum toxin for the treatment of spasticity or for deciding on the type of episiotomy during child delivery. Surface EMG (sEMG) can be noninvasively recorded to assess physiological and morphological characteristics of contracting muscles. However, it is not often possible to record signals of high quality. Moreover, muscles could have multiple IZs, which should all be identified. We designed a fully-automatic algorithm based on the enhanced image Graph-Cut segmentation and morphological image processing methods to identify up to five IZs in 60-ms intervals of very-low to moderate quality sEMG signal detected with multi-channel electrodes (20 bipolar channels with Inter Electrode Distance (IED) of 5 mm). An anisotropic multilayered cylinder model was used to simulate 750 sEMG signals with signal-to-noise ratio ranging from -5 to 15 dB (using Gaussian noise) and in each 60-ms signal frame, 1 to 5 IZs were included. The micro- and macro- averaged performance indices were then reported for the proposed IZ detection algorithm. In the micro-averaging procedure, the number of True Positives, False Positives and False Negatives in each frame were summed up to generate cumulative measures. In the macro-averaging, on the other hand, precision and recall were calculated for each frame and their averages are used to determine F1-score. Overall, the micro (macro)-averaged sensitivity, precision and F1-score of the algorithm for IZ channel identification were 82.7% (87.5%), 92.9% (94.0%) and 87.5% (90.6%), respectively. For the correctly identified IZ locations, the average bias error was of 0.02±0.10 IED ratio. Also, the average absolute conduction velocity estimation error was 0.41±0.40 m/s for such frames. The sensitivity analysis including increasing IED and reducing interpolation coefficient for time samples was performed. Meanwhile, the effect of adding power-line interference and using other image interpolation methods on the deterioration of the performance of the proposed algorithm was investigated. The average running time of the proposed algorithm on each 60-ms sEMG frame was 25.5±8.9 (s) on an Intel dual-core 1.83 GHz CPU with 2 GB of RAM. The proposed algorithm correctly and precisely identified multiple IZs in each signal epoch in a wide range of signal quality and is thus a promising new offline tool for electrophysiological studies.

## Introduction

The electromyographic signal (EMG) is the electrical activity associated to contracting muscles. The EMG signal is generated by the summed electrical activity of the muscle fibers stimulated by motoneurons [1]. This signal is recorded either invasively, known as intramuscular EMG, or noninvasively, called surface EMG (sEMG). sEMG is usually applied to asses physiological and morphological characteristics of contracting muscles and their neural strategies [2]. The use of sEMG spans from neurophysiological and medical research (aging, gait and posture analysis), to rehabilitation (biofeedback), ergonomics, sports and movement sciences (biomechanics) [3].

sEMG can be recorded using electrodes of different shapes, sizes, and arrangements [4]. Traditionally, sEMG is recorded as the potential difference between two electrodes placed on the skin, known as bipolar derivation. 1D or 2D electrode arrays including several electrodes along the lines parallel to the muscle fiber orientation are also used [5]. Such electrode systems could be used to investigate the processes of generation, propagation, and extinction of the action potentials in fusiform muscles with fibers parallel to the skin [6]. It is also possible to estimate the muscle fiber Conduction Velocity (CV) and to identify the location of the muscle Innervation Zones (IZs).

Knowledge of the location of muscle IZs in fusiform muscles with fibers parallel to the skin is important for many reasons [6]. The correct estimation of sEMG variables for the monitoring of muscular activity and the detection of muscle fatigue implies electrode positions with knowledge on the IZ location [7, 8]. Moreover, the quantity of injected botulinum toxin for the treatment of spasticity can be minimized if injected close to the IZ [9]. Optimization of neuromuscular electrical stimulation [10], motor point biopsy for diagnosis of neuromuscular diseases [7, 11], and decisions on the type of episiotomy during child delivery [12, 13] are other examples of uses of the information on IZ locations.

Manual identification of the location of muscle IZs by visual analysis [14] is a time-consuming procedure. Several methods have been proposed in the literature to automate this procedure [15–21]. Some of these methods can detect at most one IZ location in each signal interval [15, 17, 18, 20]. There are also various methods to identify multiple IZs. Masuda *et al* developed a technique based on correlation analysis [16]; the developed program was able to identify up to two IZs in good-quality recordings of biceps brachii muscle. Cescon *et al*. developed a technique based on Radon transform [21] which could detect multiple IZs in high-SNR sEMG recordings of the gracilis muscles. Mesin *et al*, proposed a method based on the matched filter [19] which was validated on 20-dB simulated sEMG signals with a maximum of two IZs. All these previous studies have focused on detecting one or two IZs on high-quality sEMG signals. However, it is not often possible to record good quality signals, particularly when using linear arrays of electrodes [3, 22, 23]. In addition, muscles have usually multiple IZs, e.g., the external anal sphincter, brachioradialis, and biceps brachii [3, 16, 24], muscle fibers in partially denervated muscles [25] and fasciculating Motor Units (MUs) in Amyotrophic Lateral Sclerosis (ALS) patients [26] may have more than one IZ as well. The aim of this paper is to automatically identify multiple IZs in medium and low-SNR sEMG signals. A method based on Graph-cut image segmentation and morphological image processing is presented. Part of this work was presented in abstract form [27].

## Materials and Methods

### Simulation

The model proposed by Farina *et al* was used to generate surface EMG signals [28]. This model is more complete in comparison with previous approaches [29]. In this model, the volume conductor was described as an anisotropic multilayered cylinder and the source was a spatio-temporal function describing the generation, propagation, and extinction of the intracellular action potential at the end-plate, along the fiber, and at the tendons, respectively. The Inter-Electrode-Distance (IED) was set to 5 mm as recommended in [30] to locate IZs. The remainder of the model parameters used in our study were in principle the same as those used by Mesin *et al* [19]. Finally, the number of active MUs in each 60-ms simulated signal interval was between 1 and 5. Signals were zero-phase digitally band-pass filtered [31] using an overall eighth-order Butterworth filter with cut-off frequencies 20 and 500 Hz.

For each MU number category (1 to 5), sEMG signals with SNR values of -5, 0, 5, 10 and 15 dB were simulated to include very low to moderate quality sEMG signals. Twenty Single-Differential (SD) channels were simulated along the muscle fiber direction and sampling frequency was 4096 Hz. Thirty frames (or images) with up to 5 IZs were simulated for each SNR value. The temporal location of the IZs was created randomly in each frame. The signal SNR for each simulated 60-ms epoch was defined as the RMS of the raw sEMG divided by the standard deviation of the added Gaussian noise, expressed in dB [32]. Thus, a total of 750 1-D linear array sEMG signals were simulated, considering five SNR values and maximum five MUs.

### Methods

**Fig 1:**
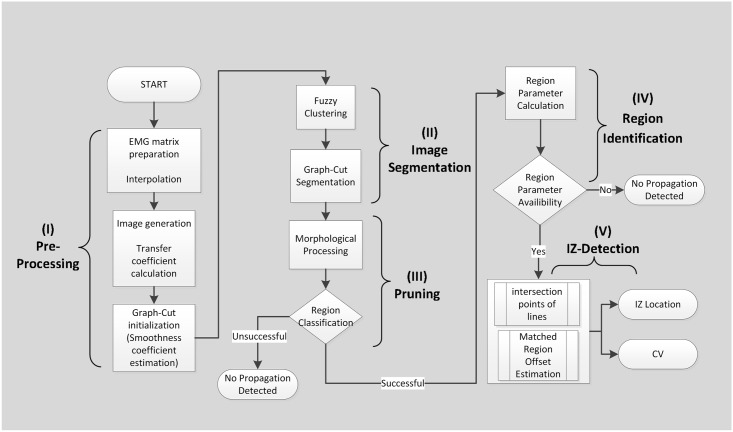
The structure of the IZ detection program including: (I) Pre-Processing, (II) Segmentation, (III) Pruning, (IV) Region identification and (V) Innervation points detection. EMG matrix preparation extracts an appropriate epoch of data for image conversion. Graph-Cut algorithm was used for image segmentation. Parameters (Slope, center/edge coordinates) in step IV were estimated to consider the interaction between regions in the image.

Fig 1 shows the flowchart of the proposed IZ detection method. The whole task breaks into 5 main parts. Detailed descriptions will be presented in the following sections; however, each part is briefly introduced as follows:

1. Pre-processing: images are generated from spatio-temporal epochs of sEMG, consisting of 1) transfer coefficient calculation to tune the relation between physical units and the pixel data needed to calculate IZ information (such as location and CV) in the time domain, and 2) initializing graph-cut image segmentation algorithm.
2. Image segmentation: this decomposes images into the specific regions related to propagations consisting of: 1) Fuzzy clustering (used as a common kernel function) and 2) Graph-cut segmentation.
3. Pruning section: a morphological technique is used as a post-processing operation to remove possible outliers.
4. Region identification: this extracts required parameters (such as coordinates of the region’s center/edge) as basic information of the IZ detection procedure.
5. IZ detection: this locates the IZ location and MU Action Potential (MUAP) CV when the image includes suitable propagating regions in which a propagation swing could be seen.

The proposed algorithm was implemented with Matlab^®^ [MathWorks^™^ Inc. Natick (MA), release 7.12.0].

#### Image generation from sEMG signals

A digital image could be defined by a 2-D *N* by *M* matrix [33], where 1 ≤ n ≤ *N* and 1 ≤ m ≤ *M* are the spatial coordinates of each pixel. The sEMG signal recorded from a linear-array of electrodes, could be considered as an image where *N* is the number of channels in the recording array, and *M* is the recorded samples in time. The activity of the muscle (amplitude of the sEMG), was normalized with respect to the maximum value of sEMG signals in the entire recording channels, to represent the intensity of each pixel as a fraction of the maximum value.

Let *E* be the original *N* by *M* sEMG data matrix containing samples sufficiently close so that both the sampling frequencies in space and in time satisfy Nyquist theorem and aliasing is absent in both directions. Interpolation between samples in space and/or in time can then be applied to obtain a new matrix *I* with dimensions *αN* and *βM*, where *α* and *β* are interpolation coefficients. Cubic spline interpolation [34] by a factor of 10 (α = β = 10) was used for both dimensions.

**Fig 2:**
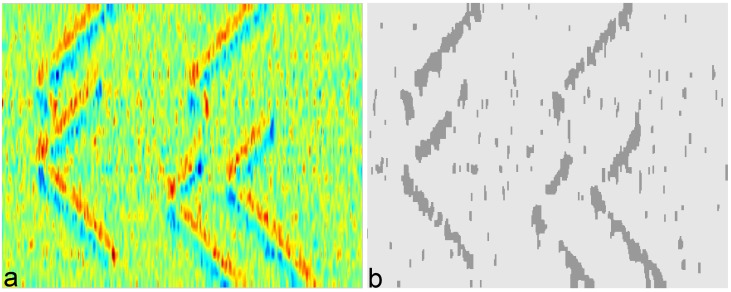
The segmentation result on a simulated sEMG with five IZs and 20dB SNR. (a) The image was generated from 60-ms epochs of linear-electrode arrays (spatial interpolation factor of 100 for visualization and the inter-electrode-distance of 5 mm) with the sampling frequency of 4096 Hz and (b) the result of image segmentation via kernel-based Graph-Cut. Propagated potentials from each IZ towards both directions (up and down in the Figure) can be seen.

In fusiform muscles with fibers approximately parallel to the skin, the amplitude of bipolar EMG signal is relatively low near the IZ locations because of potential cancellations. Propagation of MUAPs occurs from the IZ toward the muscle-tendon junctions. Thus, it creates special patterns in the corresponding regions in the image (Fig 2). By extracting such regions, it is possible to detect the IZ location as well as the MUAP conduction velocity and the direction of propagation.

In this approach, the location of IZ’s was obtained in pixel units. However it is necessary to report the results (IZ and MUAP CV) in the space and time domain. Thus a transfer coefficient was required from *E* to *I*. The calibration was performed by the algorithm to report the results in the suitable domain.

#### Image segmentation using kernel Graph-Cut

Many problems in computer vision such as segmentation, can be put into labeling problems where an undirected graph is used for data representation. It is in fact a summary representation of locations and their neighborhood structure. The solution of the labeling problem is the assignment of a label to each vertex in the graph based on the criteria in the problem’s requirements. Such criteria are represented with the energy, a cost function evaluating how good the given labeling is, where the smaller energy the better solution. Thus, the labeling problem is considered as an energy minimization problem. This function usually has many local minimums. Using graph cuts, it is possible to find the minimum of such energy functions [35]. The information about the graph-cut image segmentation method used in our study, was provided in S1 Appendix.

Let *I*: *p* ∈ Ω ⊂ *R*^2^ → *I*_*p*_ = *I*(*P*) ∈ Ψ be the image in the *R*^*2*^ space [36] defined by Ω transferred to Ψ feature space. For segmenting *I* into maximum *G* regions, each pixel must be labeled as *l ≤ G* from the finite set of labels Γ. Each region ${\left\{R_{l}\right\}}_{l=1}^{G}$ is defined as a set of pixels that have the same label. Now, λ indexing function is introduced to assign a label from the finite Γ set to each point in the image as:

λ :p∈  Ω→λ(p)∈Γ (1)

In computer vision, the segmentation is usually performed via minimizing an energy function (*F*) [37, 38]. The energy function (*F*) can then be written as:

F(λ) = D(λ) + γ R(λ) (2)

where *D* is the data energy (that encodes the constraints of the data by measuring the disagreement between the segment representative intensity and the intensity of the pixels in each segment i.e. similar to the objective or cost function of a classic clustering algorithm), *R* is the prior energy (that encodes the constraints provided by prior knowledge about the optimal labeling by measuring the extent to which labeling is not piece-wise smooth i.e. extracted segments must be smooth) and the constant γ is a positive regularized coefficient that controls relative importance of data and prior term [39]. This form of objective function is also used in Regularized Least Squares (RLS), where the first term is supposed to minimize. However, the knowledge about the possible solutions are also expressed as a penalized problem in which a regularized function (the second term) is added to the objective function [40].

In our study the regularized coefficient was adaptively estimated based on the quality of the image frame (Quality factor: QF) estimated by the method proposed by Zhang *et al* [41]. We fitted a linear regression model between QF (input) and the regularized coefficient (output) in a set of image frames with variety of SNR values. Such frames were not used for validation. The regression equation was output = 0.801×QF+0.888 (R^2^ = 0.8). The regularized coefficient ranged from 0.226 to 0.639, in our study.

According to the maximum a posterior (MAP) formulation and piecewise constant segmentation model, the data term could be defined as follows [36, 39, 42]:

$$D(\lambda )=\sum_{P\in \Omega }D_{P}(\lambda (P))=\sum_{l\in \Gamma }\sum_{P\in R_{l}}{\left(\mu_{l}-I_{P}\right)}^{2}$$ (3)

where *μ*_*l*_ is the piecewise constant model of *R*_*l*_ (i.e. the segment representative intensity). The term *D*_*P*_*(λ(P))* measures how much assigning *λ(P)* to pixel *P* disagrees with the original pixels’ intensity. The prior energy term expressing the smoothness constraints is defined as follows:

$$R(\lambda )\;=\;\sum_{\{p,q\}\in \text{ H}}r(\lambda (p),\lambda (q))$$ (4)

The entire neighboring pairs *{p*,*q}* are stored in a neighborhood set *H*. The term *r* is the smoothness regularization or neighbor interaction function, gives penalties to neighboring pixels when they have different labels. Thus the smoothness energy prior is the sum of interaction value between every pair in the neighborhood set [39]. Briefly, it measures how different the intensities of pixels are from each other in each segment.

The smoothness prior function could be obtained by the truncated squared absolute difference principle i.e. min(*const*^2^,|*μ*_*λ*(*p*)_ − *μ*_*λ*(*q*)_*|*^2^) [42]. Unlike the other functions such as mean square error and mean absolute difference, the truncated quadratic function is robust and thus it limits the influence of mismatches. This assigns higher value to the larger difference between labels and vice versa. The “*const*” term is a constant value that is used to make the function more robust and prevent possible outliers [39, 42, 43]. In the pre-processing procedure, the weight of smoothness constraint was estimated (Graph-Cut initialization). Accordingly, any abrupt changes of the image were detected and the corresponding smoothness weights were calculated.

In image segmentation, an objective function measures the goodness of the possible solutions (i.e. the label of pixels) according to a set of constrains (i.e. the prior term). The lower the value of the objective function, the better goodness of fit is reached. Thus the global minimum of the objective function gives the optimized solution[39]. In order to minimize the objective function*F(λ)*, it is useful to transform image data to the kernel space via a mapping function *φ* and to write the segmentation function in the kernel space. In machine learning, the ‘kernel trick’ is using a linear classifier to solve a nonlinear problem by mapping the original nonlinear data into a higher dimensional space. It is particularly useful to consider the complexity of image data and insufficiency of computationally efficient models such as piecewise Gaussian to partition nonlinear separable data [36]. Meanwhile, data are not handled directly and the dot product in the kernel space suffices to write the kernel induced function based on the Mercer’s theorem. The kernel function must be continuous, symmetric, and positive semidefinite. In fact, the function *φ* is not needed to be computed explicitly [36, 44, 45]. The kernel induced segmentation function could be defined as the following:

$$F_{K}(\{\mu_{l}\},\lambda )=\;\sum_{l\in \Gamma }\sum_{p\in R_{l}}J_{K}(I_{P},\mu_{l})+\gamma \sum_{\{p,q\}\in \text{ H}}r(\lambda (p),\lambda (q))$$ (5)

where *J*_*K*_ is non-Euclidean distance measure defined as:

$$J_{k}(I_{P},\mu )=\text{ ​}{\left|\left|\varphi \left(I_{p}\right)\text{​ }-\text{​ }\varphi \left(\mu \right)\right|\right|}^{2}=\;K\left(I_{p}\;,\;I_{p}\right)\;+\text{​ }K\left(\mu \;,\;\mu \right)\;-\;2K\left(I_{p}\;,\;\mu \right)\;\mu \in {\left\{\mu_{l}\right\}}_{1\leq l\leq N}$$ (6)

According to the mercer’s theorem [45], the kernel function *K(x*,*y)* can be written as:

$$K(x\;,\;y)\;=\;\varphi {\left(x\right)}^{T}.\varphi (y),\;\forall (x,y)\in \Psi$$ (7)

In our study, the Radial basis Function (RBF) was used as the kernel function (*K*). Accordingly, Eq 2 was transformed into Eq 5, in which *J*_*K*_ was calculated using Eq 6. Solving image segmentation in a kernel-induced space with graph cuts consists of finding the labeling to minimize Eq 5. In fact, the kernel induced segmentation function is used via the following optimization strategy:

We can optimize the kernel segmentation function in two steps. First, we use RBF kernel function to fix some points as region centers, by using the Fuzzy C-Mean (FCM) as to initiate the segmentation. Second, the locations of the points are optimized and upgraded by the graph-cut technique [36]. The result of such segmentations on an epoch of EMG signal is shown in Fig 2.

#### Pruning and region identification

Removing outliers (i.e. segments not related to propagation) is necessary prior to extracting features from the extracted regions. It is thus necessary to separate relevant regions and to remove overlapped/irrelevant regions. The pruning procedure consists of four steps performed by morphological processing. The detailed tutorial about the morphological image processing techniques used in our study, was provided in S2 Appendix.

**Fig 3:**
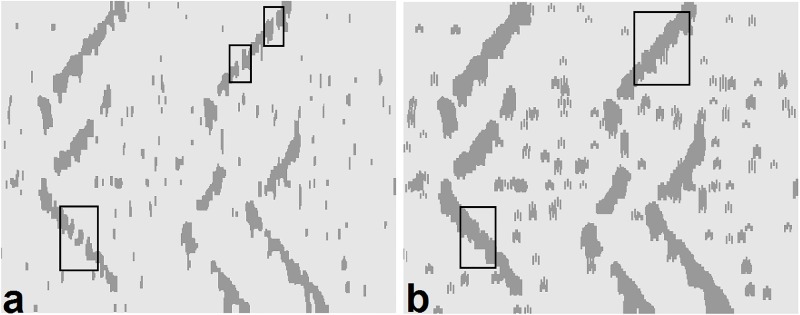
The compensation of the breakpoints of the image frames in the pruning step. (a) the segmented image with discontinuous regions (some breakpoints are shown in rectangular area). (b) The compensated image with fewer breakpoints and intensified regions.

**Fig 4:**
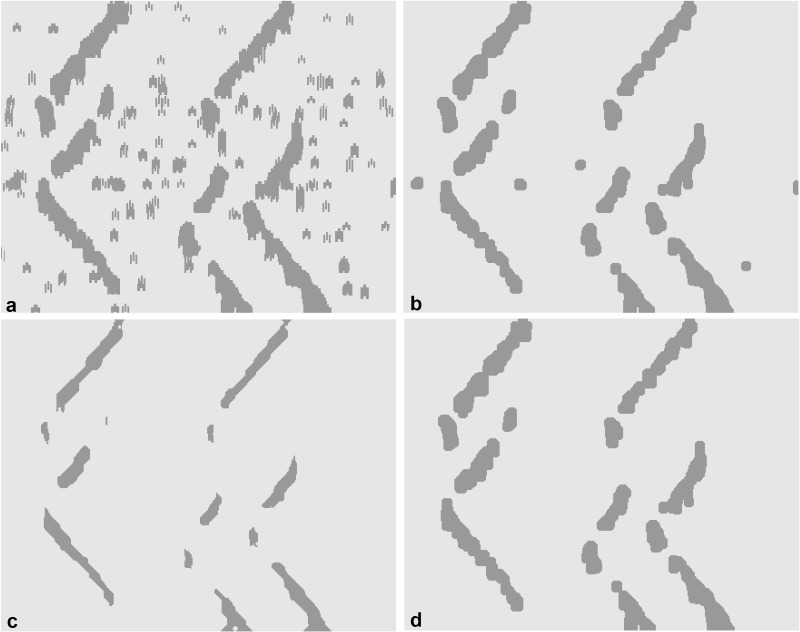
The morphological processing (pruning) steps on the result of Fig 3 consist of (a) Dilating image with pair mode structure, (b) Opening region via disk structure, (c) Region erosion via line structure and (d) reconstructing region according to the original image. In such procedures, the irrelevant structures i.e. non-propagating parts were suppressed while the detectability of the propagating regions close to the innervation zones was improved.

Morphological processing is used as a tool for extracting image components useful in a variety of machine learning problems, such as pruning [33]. We used several morphological techniques, such as dilation, by which the discontinuity of the extracted regions is compensated. The breakpoints i.e. the areas in which the propagation pattern is strongly influenced by noise are recovered via the pair-point structure (the logical OR operation between the pair structures with the offset values of [-3,3] and [3,3]) (Fig 3). Opening is used to separate the overlapped regions in order to extract symmetric regions via disk structure (the radius of 6 pixels). Then, Erosion operation is used to suppress any irrelevant regions not related to symmetric propagation via the line structure in both directions of the propagation (with the length of 20 pixels and degrees of 45° and -45° for the upper and lower V-shaped propagations, respectively). The erosion of the image was performed using these two line elements, in parallel, and the logical OR operator was used to combine these two images. Finally, such regions are reconstructed using 4-connectivity morphological reconstruction where the marker was the opened image and the mask was the resulting image after erosion. These procedures are shown in Fig 4. The extracted regions were classified as unsuccessful (i.e. no clear propagation), or successful at this step. Candidate regions are then processed at the next step. The MUAP propagation has positive and negative swings (bright and dark portions). The image segmentation is performed on both swings. For unsuccessful regions in each swing, the other swing is also analyzed and the results are then combined when successful regions are formed. The parameters of morphological structuring elements were tuned using trial and error. For the detailed information about the morphological image processing, the reader is invited to read the review on mathematical morphology by Heijmans [46].

#### IZ detection and feature extraction

**Fig 5:**
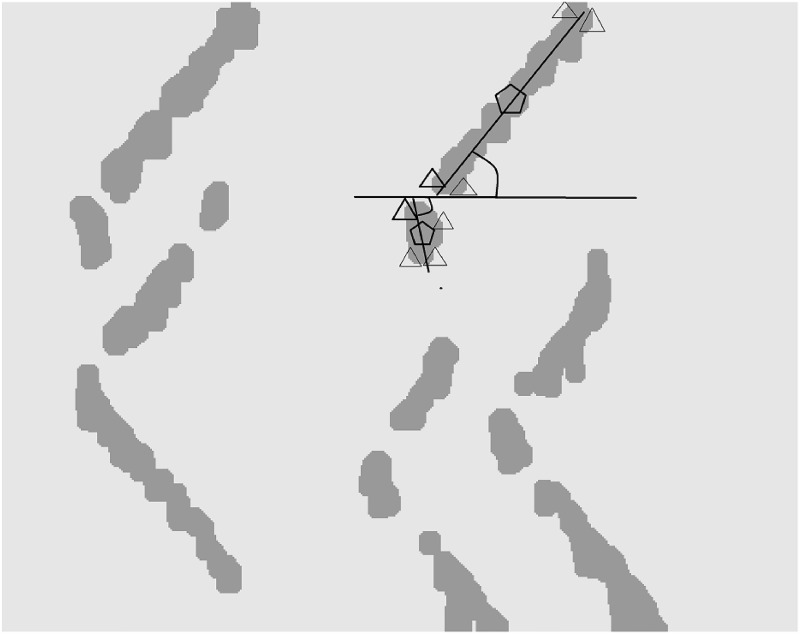
An example of the propagating region identification procedure (stage IV of the proposed algorithm) and feature extraction. The slope parameters found by center/edge coordinates are shown by triangles and pentagons, respectively. Bold triangles show the closest distance of edges. The center is defined as the center of each propagating region. The edges are the upper and lower boundaries of such regions. The slope is calculated based on the angle between a virtual line representing the propagation region (bold line) and the horizontal line.

The images generated in the previous section (Fig 4d), are directly related to the IZ and MUAP propagations. The parameters slope and center/edge coordinate are extracted for each region to perform paired-region labeling and offset calculation (Fig 5). Regions not paired in the algorithm, were excluded from further analysis. The offset of paired-region is calculated by averaging two edge coordinates of the regions with the closest distance.

**Fig 6:**
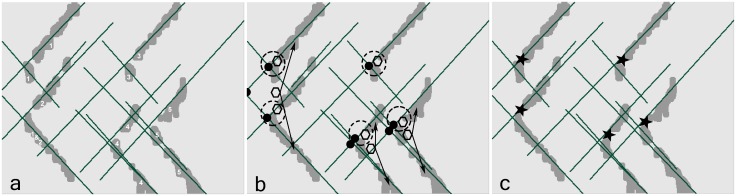
**The feature extraction procedure** including (a) assigning labels to the paired-regions (paired regions are shown with the same labels). Each region is represented by a straight line to calculate the intersection points. (b) Calculation of the offset of the paired-region and intersection of lines, offsets and intersection points are shown by hexagons and stars inside black circles respectively. (c) The estimation of the innervation zone by choosing appropriate hexagon according to its distance with corresponding intersection point. The detected innervation zones are shown in pentagon stars.

The IZ detection procedure is then performed in three steps. First, labels are assigned to each “paired-region” defined as the symmetric opposite propagation. This region is then represented with a line (Fig 6a). Second, the offset of the paired-regions and intersection of lines are calculated (Fig 6b). The intersection points and offsets of nearby paired-regions with minimum distance are linked together. This operation iterates until all points are checked. (Fig 6c; the labels with min. distances are shown by pentagon stars). Finally, MUAP conduction velocity is estimated based on the slope of the extracted propagating areas.

### Validation

Each 60-msec sEMG frame was automatically analyzed by the proposed algorithm. In each frame, if one IZ was found within ±0.5 IED (and ±0.5 msec) of a simulated IZ, it was counted as a True Positive (*TP*). When no IZ was identified within the above threshold, it was considered as a missed IZ (False Negative: *FN*). Those detected IZs in a frame not considered as *TP*s, were counted as False Positives (*FP*s). No post-processing was performed on the propagating areas to reject *FP*s if the CV was not within the normal range [47, 48]. In each SNR category, the total *TP*s, *FN*s and *FP*s were calculated in the entire 150 signals and the performance of the proposed method was assessed. The following performance measures are reported: Sensitivity (= Recall) (*Se*) as the proportion of simulated IZs correctly identified, Precision (= PPV) (*Pr*) as the proportion of identified IZs that are correct, % Missed IZs, % Erroneous IZs, and *F*_*1*_-score, defined as micro- or macro-averaging measures[49], defined as:

$$Se=\frac{TP}{TP+FN}$$ (8)

$$Pr=\frac{TP}{TP+FP}$$ (9)

% Missed IZs=100(1−Se) (10)

% Erroneous IZs=100(1−Pr) (11)

$$F_{1}-score=\frac{2\times Pr\times Se}{Pr+Se}$$ (12)

In the micro-averaging procedure, the number of *TP*s, *FP*s and *FN*s in each frame are summed up to generate cumulative measures and precision, recall and *F*_*1*_-score are then evaluated. In the macro-averaging, on the other hand, precision and recall are calculated for each frame and their averages are used to determine *F*_*1*_-score. For each correctly identified IZ, the location error measured as the absolute channel error (IED ratio), and the absolute and relative muscle fiber conduction velocity error are also reported.

To examine the effect of spatial distribution of the IZ locations on the performance of the proposed algorithm, the Average Nearest Neighbor (ANN) distance [50] was calculated in the image space for different distributions of the IZ channels. ANN was defined for each analyzed image frame as follows:

$$ANN=\frac{\sum_{i=1}^{n}d_{i}}{0.5\sqrt{n\times A}}$$ (13)

where *n* is the total number of IZ locations simulated in that frame, *A* is the total number of pixels in the image frame with the size of *αN* by *βM* (i.e.*αβ×NM*), and *d*_*i*_ is the Euclidean distance between the IZ location *i* and the closest neighboring IZ location in pixels. The lower the ANN value, the closer the IZ locations. In our study, the values of parameters *M* and *N* are (60 ms × 4096; rounded) and 20, respectively. Parameters α and β are identical to 10. Thus, the value of *A* is 492000.

On the other hand, to identify whether the closeness of the IZ locations to the frame center could affect the performance of the proposed algorithm, the spatial feature Center Closeness (*CC*) in the range of [0,1] was defined as follows:

$$CC=\frac{2\times \sum_{i=1}^{n}d_{i}^{\prime }}{n\times \sqrt{{\left(\alpha N\right)}^{2}+{\left(\beta M\right)}^{2}}}$$ (14)

where *d*_*i*_ is the Euclidean distance between the IZ location *i* and the center of the image frame in pixels. The higher the CC value, the closer the IZ locations to the frame borders.

### Statistical analysis

We aimed to identify whether the complexity of the recorded signal and the spatial information of the IZ locations could affect the performance of the proposed IZ detection algorithm. Thus, the Poisson regression model [51] was used to determine the significance of the relationship between the count data number of missed (FNs) and erroneous (FPs) (dependent variable) and the number of IZ locations in each frame, frame SNR, and spatial parameters ANN and CC (independent variables). The level of significance was set to *P*<0.05. Data was analyzed using STATA 10 [52].

## Results

**Fig 7:**
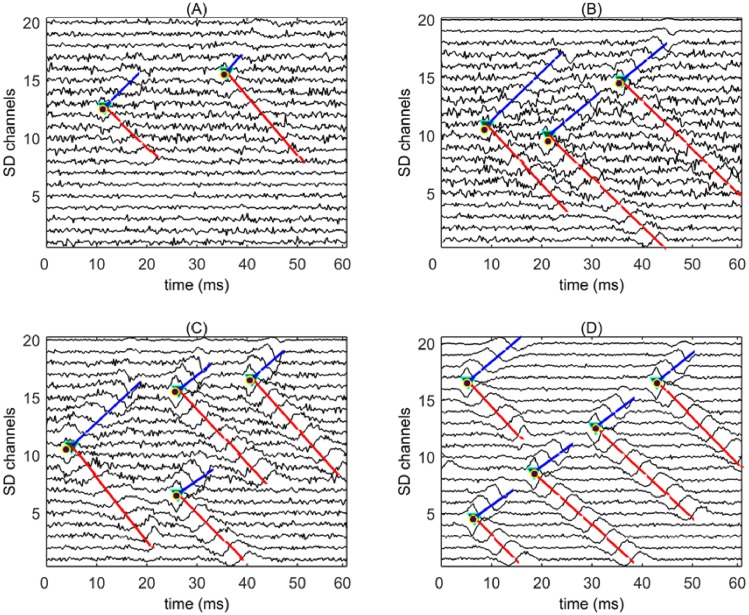
Examples of the simulated sEMG signals with 20 Single Differential (SD) channels and 60-ms epochs. The image frames A to D contained 2 IZs (-5 dB SNR), 3 IZs (0 dB), 4 IZs (5 dB) and 5 IZs (10 dB), respectively. The location of the simulated IZs is shown by circles. The developed program automatically identified the location of IZs as the crossing of the ‘v’ shape propagation lines (upper lines in blue and lower lines in red color). The CV of the identified propagation pattern was then estimated by the proposed algorithm.

Some of simulated sEMG signals are shown in Fig 7 as examples. Image frames A to D contained 2 IZs (-5 dB SNR), 3 IZs (0 dB), 4 IZs (5 dB), and 5 IZs (10 dB), respectively. The proposed algorithm could accurately locate all of the IZ locations with the average channel error of 0.07±0.05 IED.

The spatial distribution of the simulated IZ locations is shown in Table 1 in terms of ANN and CC parameters for each IZ number category. Note that ANN parameter is not defined for frames with 1 IZ.

**Table 1:** The spatial distribution parameters of the simulated EMG frames (MEAN±SD).

#IZ	1	2	3	4	5
Parameters					
ANN	-	1.16±0.18	1.38±0.19	1.40±0.28	1.37±0.34
CC	0.44±0.14	0.37±0.10	0.45±0.08	0.46±0.05	0.49±0.07

ANN: Average Nearest Neighbor Distance; CC: Center Closeness; The averaging was performed over 150 simulations in each category.

The average number of missed (FN), erroneous (FP) and correctly identified (TP) IZs in each frame is shown in Table 2 for each number of IZs and SNR category.

**Table 2:** The performance of the proposed IZ detection algorithm on the simulated dataset (MEAN±SD).

#IZ	SNR (dB)	-5	0	5	10	15
1	TP	1.0±0.0	1.0±0.0	1.0±0.0	1.0±0.0	1.0±0.0
	FN	0.0±0.0	0.0±0.0	0.0±0.0	0.0±0.0	0.0±0.0
	FP	0.0±0.0	0.0±0.0	0.0±0.0	0.0±0.0	0.0±0.0
2	TP	2.0±0.0	2.0±0.0	2.0±0.0	2.0±0.0	2.0±0.0
	FN	0.0±0.0	0.0±0.0	0.0±0.0	0.0±0.0	0.0±0.0
	FP	0.0±0.0	0.0±0.0	0.0±0.0	0.0±0.0	0.0±0.0
3	TP	2.0±1.0	2.7±0.5	3.0±0.0	3.0±0.0	3.0±0.0
	FN	1.0±1.0	0.3±0.5	0.0±0.0	0.0±0.0	0.0±0.0
	FP	0.8±1.0	0.1±0.4	0.0±0.0	0.0±0.0	0.0±0.0
4	TP	2.0±1.3	3.2±0.8	3.4±0.5	3.6±0.5	3.7±0.5
	FN	2.1±1.3	0.8±0.8	0.6±0.5	0.4±0.5	0.3±0.5
	FP	0.8±0.8	0.1±0.3	0.0±0.0	0.0±0.0	0.0±0.0
5	TP	1.5±1.2	3.2±0.8	4.3±1.1	3.8±1.1	4.5±1.0
	FN	3.5±1.2	1.9±0.9	0.7±1.1	1.2±1.1	0.5±1.0
	FP	1.3±0.9	0.5±1.0	0.2±0.4	0.3±0.5	0.3±0.7

#IZ: the number of innervation zones in each frame; TP (True Positive); FN (False Negative); FP (False positive); The averaging was performed over 30 simulations in each category.

The overall micro-averaged performance of the proposed IZ location detection algorithm for each SNR category is shown in Table 3. Overall, sensitivity, precision and F_1_-score of the algorithm on the sEMG frames with positive SNR were 92.5%, 98.3% and 95.3%, respectively. Also, the macro-averaged sensitivity, precision and F1-score of the proposed algorithm on the entire frames were 87.5%, 94.0% and 90.6%, respectively.

**Table 3:** The overall performance of the innervation zone detection algorithm.

Criteria	Sensitivity (%)	Precision (%)	Missed IZ (%)	Erroneous IZ (%)	F_1_-score
SNR (dB)					
-5	55.2	72.2	44.9	27.8	62.5
0	80.7	93.7	19.3	6.3	86.7
5	90.3	98.0	9.7	1.9	94.0
10	92.1	98.7	7.9	1.3	95.3
15	95.2	98.1	4.9	1.9	96.6

The total TPs, FNs and FPs were calculated in the entire frames in each SNR category (i.e. 150 simulations) and the micro-averaged performance indices were estimated.

For the correctly identified IZ locations, the bias error for the innervation zone detection algorithm for each IZ number and SNR category is listed in Table 4. Overall the average bias for IZ channel selection was 0.02±0.10 IED ratio.

**Table 4:** The absolute channel error (IED ratio) for the innervation zone detection (MEAN±SD).

SNR (dB)	-5	0	5	10	15
# IZ					
1	0.01±0.05	0.01±0.03	0.00±0.03	0.01±0.03	0.01±0.02
2	0.05±0.29	0.01±0.11	0.00±0.09	0.00±0.07	0.00±0.03
3	0.02±0.31	0.02±0.14	0.00±0.09	0.01±0.10	0.02±0.11
4	0.10±0.34	0.03±0.13	0.01±0.09	0.03±0.09	0.01±0.08
5	0.03±0.15	0.02±0.11	0.02±0.11	0.01±0.08	0.01±0.12

#IZ: the number of innervation zones in each frame; the error values could be transferred to mm by multiplying IED (5 mm); The averaging was performed over 30 simulations in each category.

The absolute and relative absolute muscle fiber CV error of the proposed algorithm for each IZ number and SNR category is listed in Table 5. Overall the average absolute and relative CV estimation errors were 0.41±0.40 and 11±10%, respectively.

**Table 5:** The absolute and relative muscle fiber conduction velocity error in m/s, and percentage, respectively of the proposed algorithm (MEAN±SD).

SNR (dB)	-5	0	5	10	15
# IZ					
1	0.23±0.20 (5±5)	0.28±0.25 (7±6)	0.13±0.13 (3±3)	0.26±0.25 (6±6)	0.22±0.14 (5±3)
2	0.65±0.46 (16±11)	0.39±0.30 (11±8)	0.28±0.30 (7±8)	0.42±0.32 (10±8)	0.33±0.26 (8±7)
3	0.80±0.65 (20±17)	0.59±0.51 (14±13)	0.45±0.38 (12±10)	0.51±0.39 (13±10)	0.48±0.36 (13±9)
4	0.48±0.46 (12±12)	0.32±0.36 (8±10)	0.33±0.37 (9±10)	0.31±0.36 (8±10)	0.22±0.26 (5±6)
5	0.61±0.48 (15±13)	0.56±0.45 (15±12)	0.41±0.39 (10±10)	0.44±0.43 (11±11)	0.42±0.35 (11±9)

#IZ: the number of innervation zones in each frame; the relative errors (%) are shown in parenthesis; The averaging was performed over 30 simulations in each category.

The computational complexity of the proposed algorithm was assessed in terms of the program running time on each image frame in average for each IZ number category (Table 6). Overall, the average running time of the proposed algorithm on each 60-ms sEMG frame was 25.5±8.9 (s). The program was analyzed on an Intel dual-core 1.83 GHz CPU with 2 GB of RAM.

**Table 6:** The running time of the innervation zone detection algorithm on each 60-ms sEMG frame in MEAN±SD.

# IZ	1	2	3	4	5
Running time (s)	23.4±9.1	22.5±6.3	27.7±7.4	27.3±11.3	25.6±9.1

#IZ: the number of innervation zones in each frame; The averaging was performed over 150 simulations in each category.

The regression models produced moderate fits (Pearson dispersion ratio ≈ 0.8 in both FN and FP models). The regression results showed that, all the considered parameters except CC were significant in the FN model (i.e. the number of missed IZs) while the SNR category and the number of IZs in the frame were significant in the FP model (i.e. the number of erroneous IZs) (p < 0.05). Thus, whether the IZ locations are closer to the frame border or not, does not affect the performance of the algorithm.

## Discussion

### Settings of the algorithm

For the series-fibered muscles, such as brachioradialis, up to 6 distinct endplate zones (4 zones on average) have been observed [24]. In a study performed by Cescon *et al*, about 5 to 6 different IZs were identified and further analyzed in the external anal sphincter muscles [12]. Thus, an automatic detection algorithm should address the issue of multiple IZs (Fig 7).

Among different image segmentation algorithms, Graph-cut was used in our study. It was used in low-level vision problems in the literature [53]. There is a link between this method and diffusion and it enforces piecewise smoothness while preserving relevant sharp discontinuities [54]. Watershed image segmentation, on the other hand, was used for segmentation of sEMG images to improve muscle activity estimation [55]. It is possible to combine these two techniques to improve image segmentation [56, 57], which is the focus of our future activity.

In morphological image processing steps of our algorithm, dilation was first used to fill the gaps between propagation regions. The pair-point structure was used in this step, since it reduces the probability of hits. Thus, the possibility of merging propagation regions of different IZs is reduced. Meanwhile, the detected propagation lines must span within three recording electrodes to accept the related propagation region in our algorithm.

Utilizing the IED of 5 mm in our simulations, which was also used in similar studies [16, 18], the spatial aliasing is negligible [30, 58]. In our study, the sampling frequency of 4096 Hz was used to simulate sEMG signals. Then, an interpolation factor of 10 was used to obtain the image frame. Thus, another sampling frequency could be used changing the factor accordingly.

Twenty SD channels were simulated in each array in our study. The detection algorithm does not require a long propagation line (Fig 7A) and therefore many channels. On the other hand, the number of channels influences the number of True Negatives (TNs), defined as the number of recording channels without any IZs that were not identified as IZ channels by the algorithm in each image frame. Thus, the greater the number of channels is, the greater the number of TNs. Moreover, the number of TNs is usually much higher than that of FNs or FPs in each frame. This is the reason to use performance indexes which do not depend on TNs (Eqs 8–12). They are objective performance measures in the unbalanced datasets [49]. Conversely, accuracy, which is also defined in information theory and is not used in our study, overestimates the performance of the algorithm tending to 100% with a very high number of TNs.

Finally, the SD derivation was used because it is commonly applied to detect IZs [16, 17]. In SD EMG signals, propagating potentials along the electrode array are enhanced while the non-propagating components related to power-line-interference and end-of-fiber effect are reduced[3]. Also, it is known that monopolar EMG signals contain so-called far-field potentials [59].

### Detection performance

The proposed algorithm could perfectly identify up to two IZs in frames with the whole range of SNR (Table 2). For frames with more IZs, the performance of the detection algorithm was very good under medium levels of SNR (Tables 2 and 3). The average IZ channel bias error was less than 1 mm (0.2 IED) in more than 90% of cases and only in less than 5% of the frames, the error was slightly greater than 1.5 mm (0.32 IED).

The morphological Image processing technique used in our algorithm relies on the slope of propagation. Thus, it cannot be used in pathological cases (such as ALS [26]) where the slope of propagation is close to 90°. Finally, in fusiform muscles with multiple IZs, the CV cannot be usually reliably estimated [3]. However, in the proposed algorithm the average absolute CV estimation error was only 0.41 m/s which is in the range of the expected error in real signals [60].

### Comparison with state-of-the-art

The performance of the designed algorithm was compared with that of other methods, proposed in the literature [15–17]. Such methods identified the IZ channel in the SD signal array based on the lowest RMS amplitude or highest mean frequency values (MNF), or the lowest correlation coefficient among the channels with opposite propagations.

Although such methods required a rather long stationary sEMG frame to estimate the time or frequency domain features (e.g. 250 msec) [61], much shorter epochs (e.g., 4 msec) were used to identify the IZ channels in signals with multiple IZs [16]. This epoch length is slightly less than the action potential duration in sEMG signals. Such epochs were analyzed based on the RMS and correlation analysis to find the IZ channels in the array. However, due to the low frequency resolution of the analyzed 60-msec epoch and also its lower accuracy in comparison with other methods [15], MNF criterion was not used for comparison. The value of (micro) F_1_-score measure was 17% and 25% for the RMS and correlation analysis on the positive SNR data, respectively compared with what obtained from the proposed algorithm (F_1_-score = 95%).

The matched filter method proposed by Mesin *et al* [19] had an average error of 0.3 IED (ratio) reaching values higher than 1 IED for the simulated sEMG signals with up to two IZs in each frame and SNR value of 20 dB [20]. This method is in fact the first step (i.e., template matching) of the sEMG decomposition method proposed by Gazzoni *et al* [62]. Since the approach proposed by Gazzoni *et al* relied on MUAP segmentation in each recording channel, it failed in not only very high-SNR data to some extent [20], but in low-SNR data more frequently. Image processing methods are preferred because no detection threshold is required for the MUAPs and the whole image frame is analyzed, compared with each signal.

Östlund *et al*, developed an image processing method based on Particle Image Velocimetry (PIV) to identify the location of muscle IZ [18]. Although a wide SNR range of -15 to 30 dB was analyzed in their study, only one IZ channel was simulated and then identified. The average channel error was about 0.60 IED ratio for the PIV method. Our algorithm correctly identified all of IZs in similar cases (Table 2; one IZ category) and the error was 0.13 IED ratio.

Ullah *et el*, developed an image processing method based on bi-dimensional cross correlation between the interpolated image frame and its flipped transformation [20]. That method was applied on the MUAP templates obtained by spike-triggered averaging, thus simulated sEMG frames had a very high SNR, no noise was added, and only one IZ using the results of sEMG decomposition was considered. [20]. Although this method was virtually bias free (average IZ channel error of 1.3% IED) compared with that of our method i.e. 1.8%, the inter-quartile-ratio (IQR) was 0.23 IED ratio whereas this was only 0.18 IED in our study for our entire dataset (from one to 5 IZs, SNR range of -5 to 15 dB) considering not only one IZ or very high SNR.

Soares *et al* proposed an algorithm based on morphological image processing to estimate the CV of the sEMG frames evaluated on the simulated signals with the SNR values of 12, 16, 20, 30, and ∞ (i.e. no noise) in cases with only one IZ channel yielding an average RMS error (RMSE) for the CV of 0.07 m/s [63]. In addition, Farina *et al* proposed an approach based on the modified multi-channel maximum likelihood estimation and beamforming for sEMG frames with only one IZ [60], yielded an average CV RMSE of 0.13 m/s on 12 dB SNR simulated signals[63].

On the other hand, the CV RMSE in our algorithm was 0.20 m/s for sEMG frames including one IZ with positive SNR (5, 10 and 15 dB), that is, with lower SNR considered in the previous studies.

### Additional analysis

The performance of the proposed IZ detection algorithm was further assessed considering different simulation conditions and analysis methods. In each analysis, only a simulation condition or analysis method was changed in comparison with those of what presented in this manuscript (baseline) and the performance of the algorithm was presented.

An IED of 5 mm was used in our study. However, arrays with IED of 10 mm are often used in practice. Thus, sEMG signals with 10 mm IED were simulated and the performance of the proposed algorithm was further assessed (Table 7; Scenario 1). The performance is rather lower than that of the baseline that could be because of the spatial aliasing present in sEMG signals with 10 mm IED but not 5 mm IED [30, 58].

**Table 7:** Additional analysis of the proposed IZ detection algorithms.

Indices	Micro Precision (%)	Micro Recall (%)	Micro F_1_-score	Macro Precision (%)	Macro Recall (%)	Macro F_1_-score (%)	IZ channel error (IED ratio) MEAN±SD	Absolute CV error (m/s) MEAN±SD
Scenario								
Baseline	92.9	82.7	87.5	94.0	87.5	90.6	0.02±0.10	0.41±0.40
1	84.2	83.9	84.0	88.1	87.0	87.5	0.02±0.33	0.49±0.47
2	84.8	79.7	82.2	84.7	83.0	83.8	0.10±0.19	0.61±0.66
3	86.4	82.4	84.4	86.9	85.8	86.4	0.09±0.17	0.65±0.64
4	78.1	84.1	81.0	81.8	88.3	84.9	0.08±0.20	0.72±0.69

Scenarios: Baseline: the current simulation and analysis; 1) the IED (Inter-Electrode Distance) was set to 10 mm; 2) No time dimension interpolation (β = 1; Section: Image generation from sEMG signals); 3) Sinc-based image interpolation (Lanczos-2 filter); 4) Using power-line interferences in addition with the Gaussian noise for noise modeling; The averaging was performed over 750 simulations in each error category.

In our method, the interpolation factor of 10 was used for the time dimension (β = 10). Also, the sampling frequency of 4096 Hz was used. The performance of the algorithm was assessed when no interpolation was performed in the time dimension (β = 1) (Table 7; Scenario 2). The performance of the algorithm was deteriorated in comparison with that of baseline. Thus, it is necessary to interpolate sEMG signals in the time dimension.

In our study, cubic spline interpolation was used to increase the spatial and temporal resolution of the sEMG image frames. The performance of the proposed IZ detection method was further assessed using another image interpolation method, namely as sinc-based (Table 7; Scenario 3). From sampling theory, it follows that the ideal interpolation kernel is the sinc function, i.e. ideal low-pass filter, which is of infinite extent (unbounded support). In practical applications its support is limited by using truncation. However, there is excessive ripple in the pass band and poor attenuation in the stop band (c.f. Gibbs effect) [64],resulting in poor performance in image interpolation [65]. Windowing is used to reduce the abruptness of the truncated ends and thus improve the frequency response. Among windowed-sinc filters, Lanczos filter with 2 spatial grid points was shown to be best compromise in terms of reduction of aliasing, sharpness, and minimal ringing[66].

Cubic spline, on the other hand, has only positive values which is very interesting for image processing to guarantee positive interpolation values. It is a linear, shift-invariant filter [67]. In our study, the performance of the cubic spline was better than that of Lanczos-2 filter. One reason could be that cubic spline has a favorable stop-band response [68]. It was also indicated in the literature that it is sufficient for several practical applications [69] or conditions e.g. sampling at Nyquist rate [70].

Gaussian noise is not the only disturbance present in recorded sEMG signals. Power line interferences were further simulated and added to the signal based on the method proposed by Allen [71].

**Fig 8:**
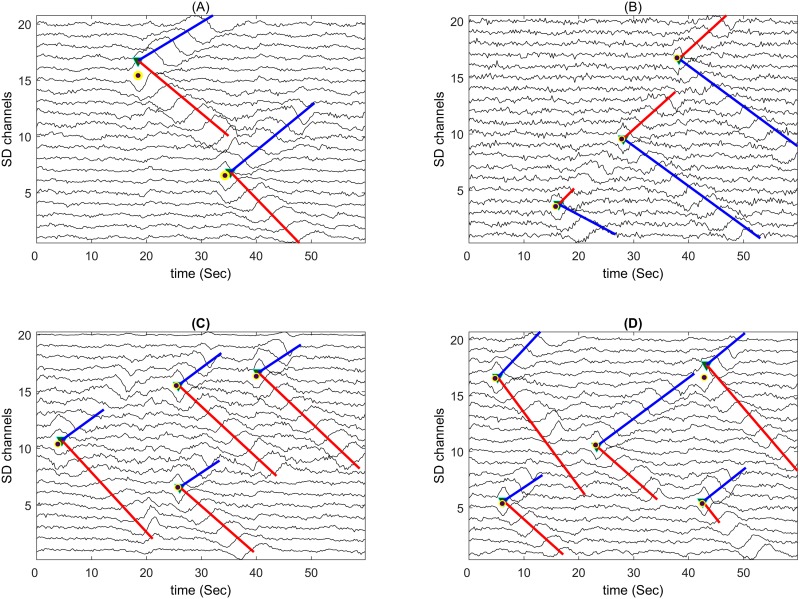
Examples of the simulated sEMG signals with 20 Single Differential (SD) channels and 60-ms epochs. The image frames A to D contained 2 IZs (-5 dB SNR), 3 IZs (0 dB), 4 IZs (5 dB) and 5 IZs (10 dB), respectively. The location of the simulated IZs is shown by circles. The developed program automatically identified the location of IZs as the crossing of the ‘v’ shape propagation lines (upper lines in blue and lower lines in red color). The CV of the identified propagation pattern was then estimated by the proposed algorithm.

The power-line interference was simulated at the fundamental power-line frequency (f_1_ = 50 Hz) and all of its harmonics up to f_10_ = 500 Hz. The frequency, amplitude and phases of each interference signal for a given sEMG were uniformly distributed to model the interference variations. The simulated main power line frequency was within 49.5–50.5 Hz. The phase distribution for the entire interference frequencies was within ±pi/2 radians. The magnitude of the fundamental frequency was determined randomly in the range 0–50% of the sEMG Root-Mean-Square (RMS) amplitude. Such ratios ranged from 25% to 50%, and 12% to 25% for frequencies f_2_, and f_3_, respectively. For the other frequencies (f_4_ to f_10_), it ranged from 6% to 12%. The Gaussian noise was then added to have the SNR range of -5 dB to 10 dB. The performance of the proposed algorithm was assessed (Table 7; Scenario 4). Its performance is rather lower than that of the baseline method. However, in practice, the power line interferences are significantly reduced when recording in SD mode. In our simulations, such interferences were directly added to the SD signals to resemble poor recording conditions. Some of simulated sEMG signals using power-line inferences in addition to Gaussian noise are shown in Fig 8 as examples. Image frames A to D contained 2 IZs (7.2 dB average SNR), 3 IZs (8.5 dB), 4 IZs (7.5 dB), and 5 IZs (6.7 dB), respectively. The proposed algorithm could accurately locate all of the IZ locations with the average channel error of 0.08±0.07 IED.

Practically, it is also possible to have intermittent contacts, whose sEMG signals are known as ‘outliers’. It is necessary to identify outliers and reconstruct sEMG time samples, prior to using our proposed IZ detection algorithm. The algorithms proposed by Marateb *et al* [23] and Ghaderi and Marateb [72] could be used. However, they require recording HDsEMG signals using 2D electrode arrays. Our algorithm could then be used for each electrode column.

### Final considerations

The type of muscle is an important factor for the IZ arrangement [18]. In fact, human muscles could have single, multiple and scattered IZs [73]. The proposed detection algorithm can be used in muscles of the first two types, since the locations of IZs are identified based on the crossing of the isolated propagation lines. In our study, simulated sEMG signals were used to evaluate the performance of the algorithm and a large variety of signal complexities was considered. Such findings could not be easily generalized to recorded sEMG signals because such signals lack the common sense of actual EMG signals [74]. However, the simulation parameters were set as to resemble fair recording conditions. Moreover, the proposed IZ detection algorithm could be used for sEMG signals with low to moderate complexity where isolated MUAPs could be seen. It requires bi-directional propagation for morphological signal processing. Also, our proposed algorithm in its current form is applicable for sEMG signals recorded with 1D electrode arrays. Using HDsEMG (High Density sEMG) signals, recorded using 2D electrode arrays, implies combining 2D spatial information and time propagation which is the focus of our future work.

## Conclusions

In conclusion, we developed a fully-automatic offline algorithm to identify multiple IZs in low-SNR multi-channel sEMG signals. In addition, CV was also estimated. This algorithm is robust and accurate and thus a promising new tool for non-invasive IZ identification. Future work will focus on the application of the algorithm to experimental signals.

## Supporting Information

S1 Appendix Image segmentation using kernel Graph-Cut. (PDF)

S2 Appendix Morphological signal processing. (PDF)
