# Supplementary material for: Detection of Multiple Innervation Zones from Multi-Channel Surface EMG Recordings with Low Signal-to-Noise Ratio Using Graph-Cut Segmentation
Source: PLoS One. 2016 Dec 15;11(12):e0167954. doi: 10.1371/journal.pone.0167954 (PMC5158322; doi:10.1371/journal.pone.0167954)
Supplement: S1 Appendix — (PDF) [file pone.0167954.s001.pdf]

Appendix I: Image segmentation using kernel Graph-Cut

Image Segmentation

A word image is used to describe a wide range of two-dimensional sensation of environment. These images may be captured by optical devices or natural object and phenomena. In computer, an image is represented numerically to display, analyze and store. In each dimension, there is a fixed number of pixels and each pixel contains a value that represents intensity (gray-scale image) or color (color image) of its location in the image [1].

In an image, there are different objects; each of which has its own area representing their nature. We may focus on a specific part of the image, based on what is required from an image. As an example, a computed tomography image contains different organs. When a specific organ, located in a specific region in the image, is needed to analyze, the related region must be extracted. Thus, segmenting an image into the sub-set of regions, it is possible to better analyze such regions in details [1].

A region is defined as a group of pixels having similar border and a specific shape like circle. The term image segmentation is referred to as the partitioning of an image into a set of regions covering it. When the regions of interest do not cover the whole image, the segmentation is used to decompose the image into foreground regions of interest and background regions to be ignored. In our problem, the segmentation of an image frame results in different propagation regions and noise areas. The former, will be enhanced and later is suppressed using morphological signal processing discussed in the Appendix 2.

Usually, segmentation has two objectives. One of which is to decompose the image into parts for further analysis, while the other aim is to perform a change of representation by increasing the efficiency of underlying regions. Such regions must be uniform and homogeneous whose interiors do not have many small holes and boundaries are smooth. Here we focus on gray scale images where the pixels contain the intensity values.

Clustering

A common cost (objective) function, minimized through clustering, is formed based on the least squares error measure:

$$D = \sum_{k=1}^{K} \sum_{x_i \in C_k} \left\| x_i - m_k \right\|^2 \qquad (1)$$

where K is the number of clusters $\{C_1, \ldots, C_K\}$, m_k is the representative of the cluster no. k (e.g. the average of the values of the samples in that cluster), and D is the cost function. Overall, it measures how the data are close to their assigned clusters. In most of the algorithms, the parameter K is set i.e. different number of clusters are tested and the optimal number is estimated based on some criteria. When this method is applied for image segmentation, connected component labeling is further used to find connected regions in each segment.

Different clustering methods such as K-means were proposed in the literature based on the minimization of the above cost function. Such algorithms, are iterative and the do not guarantee to find the global minimum of the cost function. In fact, there are many methods proposed in the literature for image segmentation (**Figure A1-1**) [2].

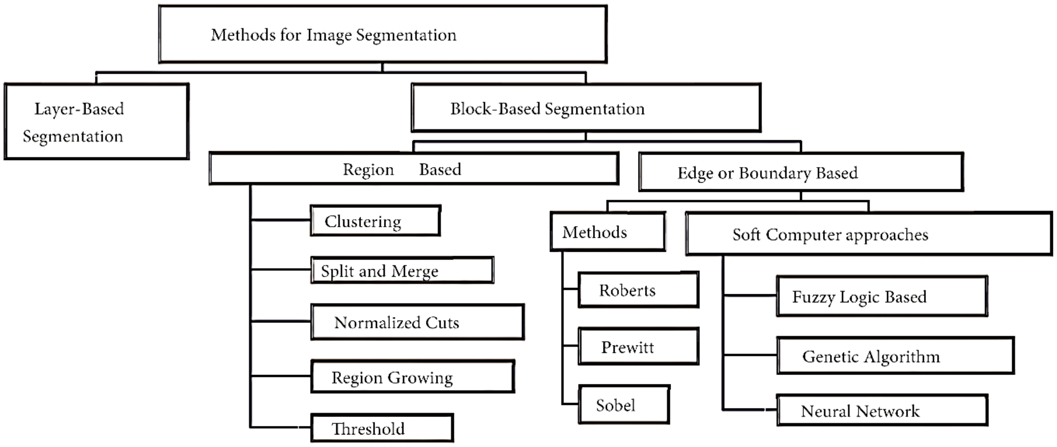

Figure A1-1: Methods for image segmentation (reproduced from [2] with permission)

Graph-cut segmentation, is one of the categories of normalized cuts in which the segmentation is based on the discontinuities in the image. This method, aimed at optimal splitting by reducing the number of regions. It is based on the graph theory in which each pixel is a vertex and edges link nearby pixels (4-connectivity) [2]. Proper image segmentation, implies defining right partition cost function and incorporating an efficient optimization algorithm. The former issue is discussed first and the energy cost function is introduced. The solution of this optimization is then provided by using the graph theory.

The energy (cost) function

For segmenting the input gray-scale image I (that has the pixel set Γ) into maximum G regions, the label of each pixel $l \leq G$ must be provided. Each region $\{R_l\}_{l=1}^{G}$ is thus defined as a set of pixels with the same label. The mapping function that indicates the label of each pixel is shown as λ.

In computer vision, the segmentation is usually performed via minimizing an energy function (F) [3, 4]. The energy function (F) can then be written as:

$$F(\lambda)=D(\lambda)+\gamma R(\lambda) \quad (2)$$

where D measures the disagreement between the segment representative intensity and the intensity of the pixels in each segment (Equation 3) i.e. similar to the objective or cost function of a classic clustering algorithm shown in Equation 1), R is the penalty function for extracted segments that are not smooth. Briefly, it measures how different the intensities of pixels are from each other in each segment. This could be estimated based on the sum of the squared difference of each pairs of pixels intensity in each segment. The constant γ is a positive regularized coefficient that controls relative importance of the first and second terms [5].

$$D(\lambda)=\sum_{l\in\Gamma}\sum_{P\in R_l}(\mu_l-I_P)^2 \quad (3)$$

where μ_l is the intensity representative of R_l (e.g. the average of the intensity values of the pixels in segment l). The cost function shown in Equation 2 could also be minimized by the transformation of the first term of Equation (2) to higher dimensions where the nonlinearity of the problem is reduced [6]. The kernel induced segmentation function could be defined as the following:

$$F_K(\{\mu_l\},\lambda)=\sum_{l\in\Gamma}\sum_{p\in R_l}\left(K\left(I_p,I_p\right)+K\left(\mu_l,\mu_l\right)-2K\left(I_p,\mu_l\right)\right)+\gamma R(\lambda) \quad (4)$$

Where K is the Radial basis Function (RBF) defined as $K(X,Y)=\exp\left(-\|X-Y\|^2/\sigma^2\right)$ where σ was set to 0.5 in our study. Transforming the data to the higher dimension is used in the literature in different applications.

Graph Cut

The cost function mentioned in equation (4) is minimized with an iterative two-step optimization strategy. The procedure is similar to other partitioning clustering (such as K-means). First, the labels are

fixed and the intensity of segments' representatives $\{u_1,\ldots,u_G\}$ is estimated using the gradient descent algorithm[6]. Second, the optimal labeling is determined by using the graph-cut algorithm. The algorithm iterates these two steps until convergence.

Let $\vartheta = \langle \nu, \varepsilon \rangle$ be a weighted graph where ν is the set of vertices (i.e. pixels) and ε is the set of edges linking nearby pixels (i.e. connection with up, down, right and left pixels). There are also two additional distinguished vertices in $\mathcal{V}$ known as terminals (source: "S" and sink "T"). A cut $\varsigma \subset \varepsilon$ is defined as a partition of vertices into two disjoint subsets in the induced graph $\vartheta(\varsigma)$ where terminals are separated in the graph [5, 7] (**Figure A1-2**). This cut is also minimal, in a sense that that none of its subsets separates the terminals into the same two sub graphs.

The cost of the cut ς is defined as the sum of its edges weights (or capacity). The problem is to find the cut with smallest cost. By setting the weight of graph ϑ based on the information of the pixels in the image, the minimum cost cuts corresponds with the minimum of the energy function. The calculation of the graph weights based on the image information is straight forward [6, 8]. Unlike other partition method where the image is first segmented into background and foreground images and the procedure continuous until no further segment is found. The method used in our study is capable of the identification of multiple regions simultaneously. This method improves the boundary of identified segments in each iteration (**Figure A1-3**).

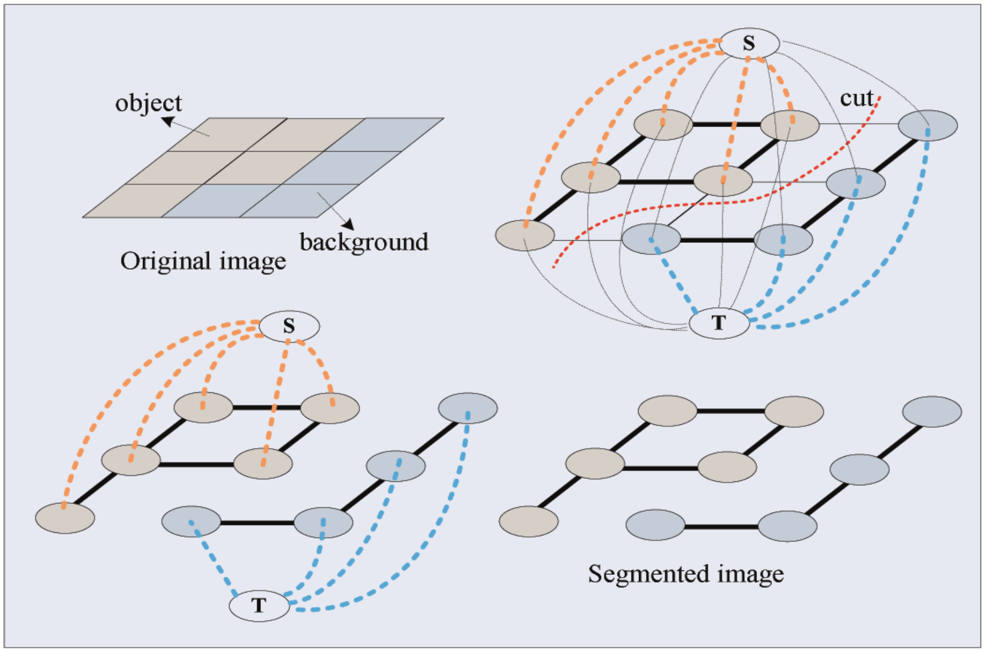

Figure A1-2: Illustration of graph cut for image segmentation. The cut is corresponding to the minimal energy of Equation 4. “S” and “T” are the terminal vertices source and sink, respectively (reproduced from [2] with permission).

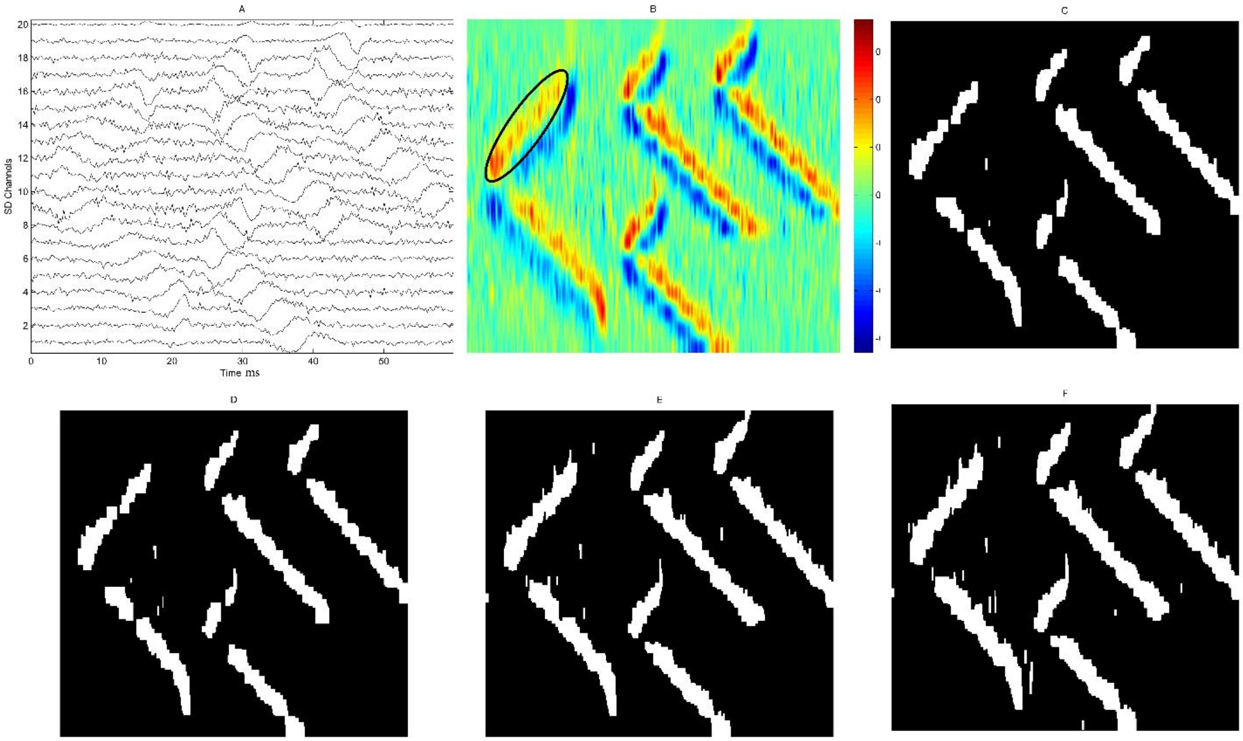

Figure A1-3: Kernel graph cut procedure for segment image. A) The original surface electromyographic signals (SNR 5dB); B) The transformed image using bi-cubic interpolation (factor of 10). The segmentation is perfumed in the positive swings in this figure (a sample region was marked by a black oval); the first, second, third and fourth iterations are shown in B-F. The boundaries of the identified segments are improved at each iteration.

References

1. Shapiro, L.G. and A. Rosenfeld, *Computer vision and image processing*. 1992, Boston: Academic Press. xiv, 623 p.
2. Zaitoun, N.M. and M.J. Aqel, *Survey on Image Segmentation Techniques.* Procedia Computer Science, 2015. **65**: p. 797-806.
3. Hofmann, T., J. Puzicha, and J.M. Buhmann, *Unsupervised texture segmentation in a deterministic annealing framework.* Pattern Analysis and Machine Intelligence, IEEE Transactions on, 1998. **20**(8): p. 803-818.
4. Poggio, T., V. Torre, and C. Koch, *Computational vision and regularization theory.* Nature, 1985. **317**(6035): p. 314-319.
5. Veksler, O., *Efficient Graph-Based Energy Minimization Methods in Computer Vision*. 1999, Cornell University.
6. Salah, M.B., A. Mitiche, and I.B. Ayed, *Multiregion Image Segmentation by Parametric Kernel Graph Cuts.* Image Processing, IEEE Transactions on, 2011. **20**(2): p. 545-557.
7. Salah, M.B., A. Mitiche, and I.B. Ayed, *Multiregion image segmentation by parametric kernel graph cuts.* IEEE Transactions on Image Processing, 2011. **20**(2): p. 545-557.
8. Boykov, Y.Y. and M.-P. Jolly. *Interactive graph cuts for optimal boundary & region segmentation of objects in ND images*. in *Computer Vision, 2001. ICCV 2001. Proceedings. Eighth IEEE International Conference on*. 2001. IEEE.
