# Supplementary material for: Detection of Multiple Innervation Zones from Multi-Channel Surface EMG Recordings with Low Signal-to-Noise Ratio Using Graph-Cut Segmentation
Source: PLoS One. 2016 Dec 15;11(12):e0167954. doi: 10.1371/journal.pone.0167954 (PMC5158322; doi:10.1371/journal.pone.0167954)
Supplement: S2 Appendix — (PDF) [file pone.0167954.s002.pdf]

Appendix II: Morphological signal processing

The word morphology is related to a branch of biology to study of the structure of organisms. Mathematical morphology, on the other hand, is a tool that could be used to extract image components such as boundaries [1]. Such techniques are also used for pre- or post-processing to improve the quality of the images and the segmentation procedures [2]. Here, we describe morphological image processing techniques used for innervations zone (IZ) detection. For simplicity, such techniques are described in binary images based on binary sets. However, the extension of binary morphological concepts to gray-scale images is pretty straightforward by replacing set union and intersections with maxima and minima [3].

The output of the Graph-cut segmentation method is used as the input to the morphological image processing algorithm. This algorithm, includes different steps to improve the image quality (**Figure A2-1**). The tutorial prepared here includes the complete Matlab functions and data to replicate the results shown in **Figure A2-1.** The required data and Matlab functions could be downloaded from https://figshare.com/articles/surface_electromyographic_data_simmulation/4212123. The Matlab ® release 7.12.0 was used for the tutorial and the Image Processing Toolbox is required for running the examples.

Preliminaries

Let A be a set in Z^2, whose elements are pixel coordinates (x,y). An element of A, $\omega = (x, y)$ is shown as $\omega \in A$ $\omega \epsilon A$.When it is not an element of A, we use $\omega \notin A$. A set B whose pixel coordinates satisfy a condition, is written as $B = \{\omega \mid condition\}$. For example, the complement of A is defined as $A^c = \{\omega \mid \omega \notin A\}$. The following three set operations are usually used in the definition of morphological image processing methods:

The difference of set *A* and *B*, the set of entire elements belonging to A not to B is defined as:

$$A - B = \{\omega \mid \omega \in A, \omega \notin B\} \quad \text{(Equation 1)}$$

The reflection of set *B*, is defined as:

$$\hat{B} = \left\{\omega \mid \omega = -b, \quad for \quad b \in B\right\} \quad \text{(Equation 2)}$$

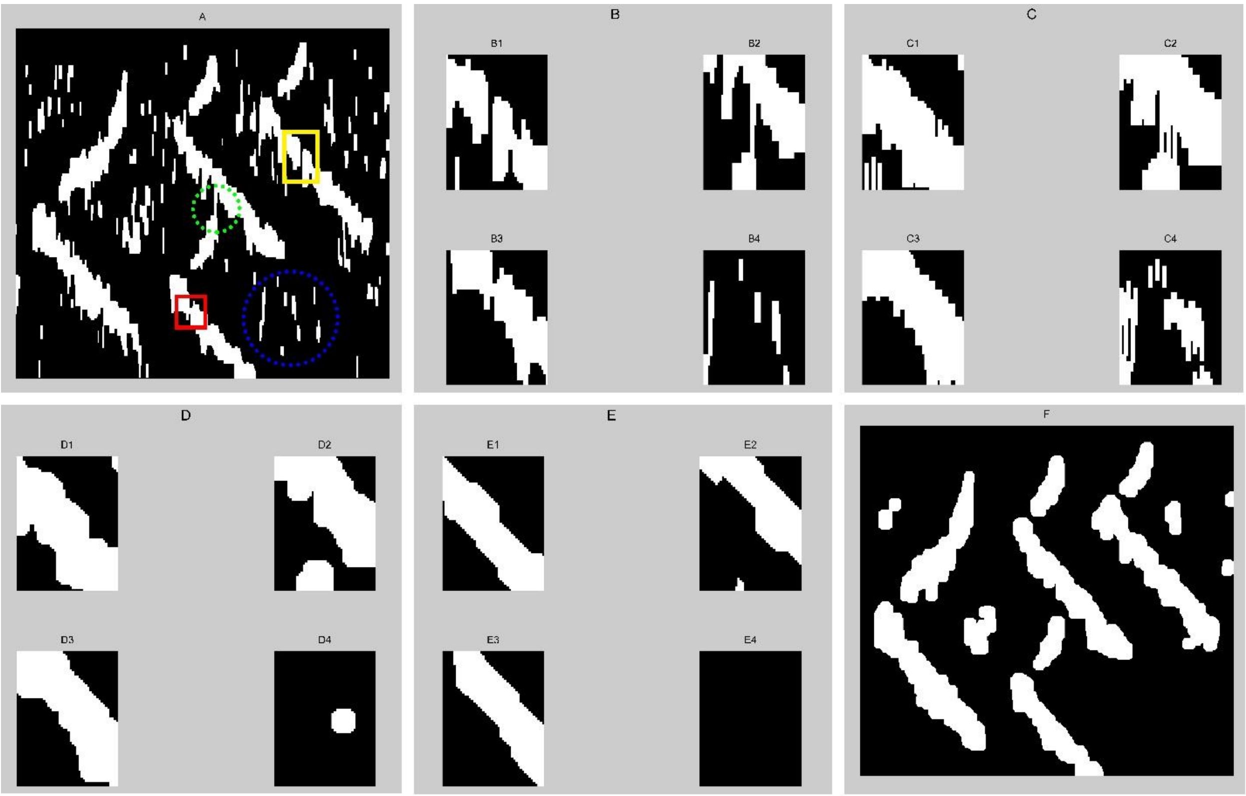

Figure A2-1: morphological binary image processing. A) The original image generated after Graph-cut segmentation. Some areas were marked as examples and further zoomed in subplot B to describe the morphological operations. Yellow rectangular shows bridge-gap on the desired region (B1). Green dotted circle shows undesired connection between two propagation regions (B2). Red rectangle shows weak (thin) propagation area (B3) while blue dotted circle shows regions related to noise (B4). C) Dilation via Pair-Point structuring element to fill the bridge gaps (C1) and to thicken weak areas (C3). D) Opening image via disk structure to smooth the regions' boundary (D1, D3) and to separate mixed regions (D2). E) Erosion using line structure to remove noise regions (E4). F) The final image after performing morphological image processing. The underlying areas (1-4) in each subplot (B-

E) are repeated to shown the effect of each morphological operation, consecutively. The size of such areas is 61 pixels (y-axis) by 46 pixels (x-axis). The size of the entire image frame (A or F) is 343 pixels (y-axis) by 365 pixels (x-axis).

The translation of set *A* by point $z=(z_1,z_2)$, is defined as:

$$(A)_z=\left\{c=a+z,\quad for\quad a\in A\right\}\qquad \text{(Equation 3)}$$

A binary image is a function of *x* and *y* i.e. $C(x,y)$, that can take either 0 or 1 [3].

When a structuring element (i.e. a binary image with rather lower dimension) is placed in a binary image, it is said to “fit” the image if, for each of its pixels set to 1, the corresponding image pixel is also 1. Similarly, a structuring element is said to “hit”, or intersect, an image if they overlap, i.e. at least for one of its pixels set to 1 the related image pixel is also 1 [4-6]. Three morphological image processing techniques used in our study (Dilation, Opening and Erosion) are then described below:

Dilation

Dilation is an operation for “growing” or “thickening” objects in a binary image (A). The procedure is controlled by a structuring element (B) which is basically a matrix of zeroes and ones. The dilation of A by B, is mathematically defined as:

$$A\oplus B=\left\{z\,|\,(\hat{B})_z\cap A\neq\varnothing\right\}\qquad \text{(Equation 4)}$$

where $\varnothing$ is an empty set and *B* is the structuring element. In our study, the logical OR operation between pair mode structuring elements with the offset values of $[-3,3]$ and $[3,3]$ was used for dilation (**Figure A2-2**). The logical OR was used to incorporate both the positive and negative swings in propagation regions.

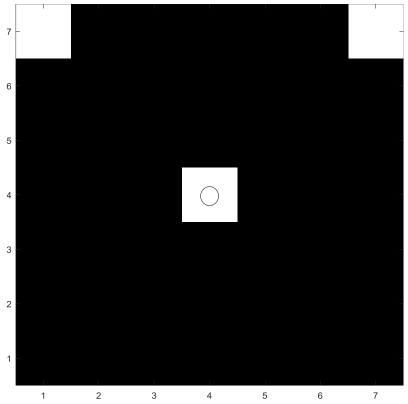

Figure A2-2: the pair mode structuring element used for dilation. The matrix includes values of 1 (white) and black (0). The origin of this element, was marked with the circle. This structuring element has, in fact, only three ones, one at the origin and the others at the upper right and left corners.

The dilation of *A* by *B* is the set consisting of the entire original locations of the structuring element in which the reflected and translated *B* overlaps. It generates a new binary image with ones in the entire locations (*x*,*y*) of an origin of a structuring element where that structuring element *B* “hits” the input image *A* and 0 otherwise, repeating for all pixel coordinates *(x,y)* [5].

The Dilation of binary images B1-B4 (**Figure A2-1**) via pair-point structuring element (**Figure A2-2**), is shown in images C1-C4 (**Figure A2-1**), respectively. When the data “morphology.mat” is loaded into Matlab, the following Matlab code could be used to reproduce images C1-C4 from binary images B1-B4.

Opening and Erosion

Opening is a morphological operator used for smoothing the object contour and breaking narrow isthmuses (i.e. separating mixed regions). The opening set A by structuring element B, is defined as below.

$$A \circ B = \left(A \ominus B\right) \oplus B \quad \text{(Equation 5)}$$

where ⊖ is the erosion operator, defined based on the duality principle as:

$$A \ominus B = \left(A^c \oplus \hat{B}\right)^c \quad \text{(Equation 6)}$$

Matlab Code **A2-1**: Dilating binary images

```
function [C,C1,C2,C3,C4]=dilate_image(A)
% Dilating binary image (A) to generate
% image C and its underlying frames C1,...,C4

% Pair mode structuring element
se= strel('pair',[3,3]);
pair = getnhood(se);
pair(7,1)=1; % Combining it (OR) with  another
% pair structure with paramters:[-3,3]
se_dilation = strel('arbitrary',pair);
% Dilation
C= imdilate(A,se_dilation);
% Creating underlying image frames
C1=C(100:160,260:305);
C2=C(140:200,174:219);
C3=C(260:320,150:195);
C4=C(240:300,240:285);
```

Erosion is the opposite of dilation, that intends to shrink or “thin” objects in a binary image. For each pixel in *A* the origin of *B* is superimposed, if *B* is completely contained by *A* the pixel is retained, else deleted (i.e. is set to zero).

The binary images obtained in the previous section, was used for opening (binary image *A*) and the disk mode structure with the radius of 6 pixels was used as the structuring element (*B*). The opening element is shown in **Figure A2-3**.

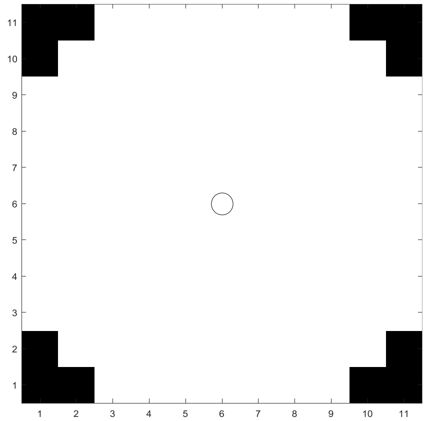

Figure A2-3: the disk mode structuring element used for opening. The matrix includes

values of 1 (white) and black (0). The origin of this element, was marked with the circle.

The opening of binary images C1-C4 (**Figure A2-1**) via disk structuring element (**Figure A2-3**), is shown in images D1-D4 (**Figure A2-1**), respectively. Loading “morphology.mat” into Matlab, and then running the function “dilate_image.m”, the following Matlab code could be used to reproduce images D1-D4 from binary images C1-C4. Using opening image, the boundary of regions C1 and C3 was smoothed (D1, and D3) and mixed propagation regions in C2 was separated (D2) (**Figure A2-1**).

Matlab Code **A2-2**: Opening binary images

```
function [D,D1,D2,D3,D4]=open_image(C)
% Opening binary image (C) to generate
% image D and its underlying frames D1,...,D4

% Disk mode structuring element
se_open= strel('disk',6);
% Opening
D= imopen(C,se_open);
% Creating underlying image frames
D1=D(100:160,260:305);
D2=D(140:200,174:219);
D3=D(260:320,150:195);
D4=D(240:300,240:285);
```

The remaining noise/unwanted regions were further removed from the images using the erosion morphological operator. Here, the line structuring element was used as to make the regions ready for the region identification step. We designed two line elements with the length of about 20 pixels, namely as positive and negative slope lines of 45° and -45°. They are shown in **Figure A2-4**. The erosion of binary image was performed using these two line elements, in parallel, and the logical OR operator was used to combine these two images.

The erosion of binary images D1-D4 (**Figure A2-1**) via line structuring element (**Figure A2-4**), is shown in images E1-E4 (**Figure A2-1**), respectively. Loading “morphology.mat” into Matlab, and then running the function “dilate_image.m” and “open_image”, the following Matlab code could be used to reproduce images E1-E4 from binary images D1-D4. Using image erosion, noise region in D4 was supposed (**Figure A2-1**).

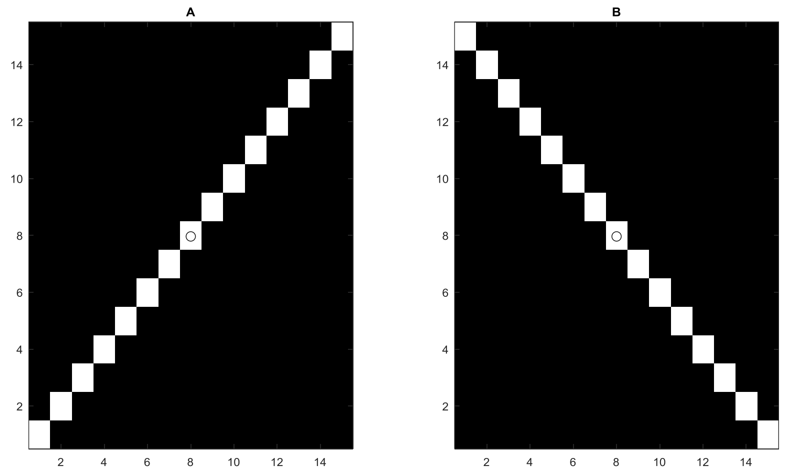

Figure A2-4: the line mode structuring element used for erosion. The line elements have the length of about 20 pixels and the positive and negative slope lines of 45˚ (A) and -45˚ (B). The matrix includes values of 1 (white) and black (0). The origin of these elements, was marked with the circle.

Matlab Code **A2-3**: Erosion of binary images

```
function [E,E1,E2,E3,E4]=erose_image(D)
% Erosing binary image (D) to generate
% image E and its underlying frames E1,...,E4

% line mode structuring element
se_eros_P = strel('line',20,-45);
se_eros_N=strel('line',20,45);

%Image Erosion
E_1=imerode(D,se_eros_P);
E_2=imerode(D,se_eros_N);
E=E_1|E_2;

% creating underlying image frames
E1=E(100:160,260:305);
E2=E(140:200,174:219);
E3=E(260:320,150:195);
E4=E(240:300,240:285);
```

Morphological Reconstruction

Using the previous morphological techniques, the bridge gaps inside a propagating region are filled (**Figure A2-1**: C1 compared with B1), the noise areas are suppressed (**Figure A2-1**: E4 compared with B4) and mixed propagation segments are disconnected (**Figure A2-1**: D2 compared with B2). However, the margins of some propagation areas are suppressed (**Figure A2-1**: E2 compared with B2). This is the usual problems, occurs, using morphological operators in which "erosion" is used. However, morphological reconstruction was proposed in the literature to resolve such a problem [3, 4].

Such a reconstruction, involves two images and a structuring element. The first image, the marker, is used as the initial image for transformation, while the other image, the mask, limits the transformation. The structuring element defines connectivity. In our study, 4-contectivity was used as the structuring element (**Figure A2-5**). The marker was the opened image while the mask was the resulting image after erosion.

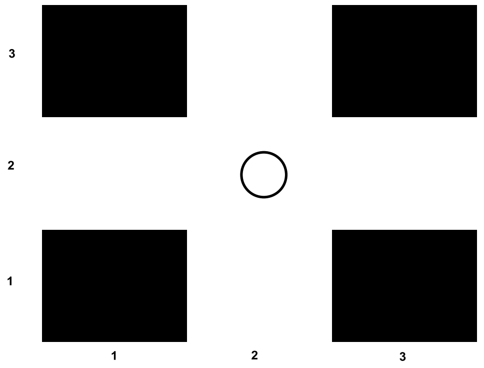

Figure A2-5: the structuring element (4-connectivity) used for morphological reconstruction. The matrix includes values of 1 (white) and black (0). The origin of these elements, was marked with the circle.

The morphological reconstruction algorithm is listed as below [3]. In this algorithm g, f, B are the mask, marker and structuring element, respectively and it is assumed that $f \subseteq g$. The related Matlab command for 4-connectivity reconstruction is "h = imreconstruct(f,g,4);"

1. Initialize h_1 (h_1=f)
2. Repeat

$$h_{k+1} = \left(h_k \oplus B\right) \cup g$$

Until $h_{k+1} = h_k$.

The entire procedure for morphological processing of binary image A (**Figure A2-1**), is shown in the following Matlab code. The resulting binary image is shown in image F (**Figure A2-1**).

Matlab Code **A2-4**: The complete morphological processing procedures

```
function F=IMP(A)
% Morphological Processing of Image A to generate final image F

% Dilating image A
[C,~]=dilate_image(A);

% Opening image C
[D,~]=open_image(C);

% Erosion of image D
[E,~]=erose_image(D);

% morphological Reconstruction of image E
mask=logical(D);
F=imreconstruct(E,mask,4);
```

References

1. Goutsias, J., L. Vincent, and D.S. Bloomberg, *Mathematical morphology and its applications to image and signal processing*. Vol. 18. 2006: Springer Science & Business Media.
2. Gonzalez, R.C. and E. Richard, *Woods, digital image processing.* ed: Prentice Hall Press, ISBN 0-201-18075-8, 2002.
3. Gonzalez, W. and R.E. Woods, *Eddins, Digital Image Processing Using MATLAB.* Third New Jersey: Prentice Hall, 2004.
4. Gonzalez, R.C. and R.E. Woods, *Digital Image Processing (2ed Edition)*. Secend ed. Vol. 1. 2001, New jersey: Prentice-Hall, Inc. 793.
5. Heijmans, H.J.A.M., *Mathematical Morphology: A Modern Approach in Image Processing Based on Algebra and Geometry.* SIAM Review, 1995. **37**(1): p. 1-36.
6. Efford, N., *Digital image processing : a practical introduction using Java*. 2000, Harrow, England ; New York: Addison-Wesley. xxiii, 340 p.
